# Introducing chaos behavior to kernel relevance vector machine (RVM) for four-class EEG classification

**DOI:** 10.1371/journal.pone.0198786

**Published:** 2018-06-29

**Authors:** Enzeng Dong, Guangxu Zhu, Chao Chen, Jigang Tong, Yingjie Jiao, Shengzhi Du

**Affiliations:** 1 Tianjin Key Laboratory For Control Theory & Applications in Complicated Systems, Tianjin University of Technology, Tianjin, The People’s Republic of China; 2 Xi’an Modern Control Technology Research Institute, Xi’an, Shaanxi Province, The People’s Republic of China; 3 Department of Electrical Engineering, Tshwane University of Technology, Pretoria, South Africa; Pablo de Olavide University of Seville, SPAIN

## Abstract

This paper addresses a chaos kernel function for the relevance vector machine (RVM) in EEG signal classification, which is an important component of Brain-Computer Interface (BCI). The novel kernel function has evolved from a chaotic system, which is inspired by the fact that human brain signals depict some chaotic characteristics and behaviors. By introducing the chaotic dynamics to the kernel function, the RVM will be enabled for higher classification capacity. The proposed method is validated within the framework of one versus one common spatial pattern (OVO-CSP) classifier to classify motor imagination (MI) of four movements in a public accessible dataset. To illustrate the performance of the proposed kernel function, Gaussian and Polynomial kernel functions are considered for comparison. Experimental results show that the proposed kernel function achieved higher accuracy than Gaussian and Polynomial kernel functions, which shows that the chaotic behavior consideration is helpful in the EEG signal classification.

## Introduction

Brain-Computer Interface (BCI) is an interdisciplinary cutting-edge technology that establishes communication and control channels between human brain and an external computer or other intelligent electronic equipment [1–5]. Motor imagery (MI) based BCIs focus on converting the recorded electroencephalograph (EEG) during imagining limb or body movements, the so-called ‘idea’, into specific codes or commands to detect EEG signal behaviour or control the intelligent equipment [6–9].

To accurately classify or decode EEG signals in BCI, pattern recognition is a vitally important step. A few EEG classification algorithms were proposed, for example, the linear discriminant analysis (LDA), the artificial neural networks (ANN), and the support vector machine (SVM), etc. The LDA [10, 11] is a two-class classification which divides the input space into two subspaces by mapping the multidimensional input vector to a hyperplane, each subspace representing one class. It was proposed that appropriate regularization of LDA by shrinkage improves the LDA performance in single-trial ERP classification [10]. The ANN [12–15] is an artificial multi-layer “neuron” inspired by the biological neuronal structure in the human brain. In ANN, a hyperplane used for classification is obtained by computing the weighted sum between neurons. Three types of ANN structures for two-class 2-D cursor movement classification were developed in [12]. A filter based on ANN [13] was proposed to reduce EEG interference signals. The SVM finds the classification hyperplane, in which two-class samples exhibit the largest distance. When the two classes are not linearly separable, the linear SVM fails to solve the classification problems. In these cases, kernel functions can be designed to map nonlinear separable samples into a high-dimensional space first, so that the samples in the high- dimensional space become linearly separable. In recent years, successful BCI experiments were reported based on SVM or its variations [12, 16–23]. For instance, a SVM method was developed to distinguish seizure EEG epochs from normal ones [21]. A hierarchical SVM algorithm was proposed for four-class EEG signal classification [23].

With further understanding of SVM, researchers gradually found some inherent shortcomings: 1. the kernel functin for nonlinear classification must meet Mercer’s condition; (i.e. for a real-valued function K(x, y), *∫∫* *g*(*x*)*K*(*x*, *y*)*g*(*y*)*dxdy* ≥ 0 for all square integrable functions g(x)) 2. the experimental results are usually sensitive to the penalty factor, which can easily lead to overfitting; 3. the output is not always reliable, and so on. Based on the Bayesian framework, the relevance vector machine (RVM) was proposed, which is similar to SVM, but overcomes these shortcomings. RVM is actually a Bayesian sparse kernel method for regression problems and classification problems [24, 25], because the final result is only related to a few relevant input vectors [26–32]. Comparing with SVM, the kernel function of RVM does not need satisfying the Mercer’s condition, so more options of kernel function can be considered. Comparing with ANN, RVM is based on small sample data to obtain the optimal solution, while ANN generally needs a large amount of training data. In motor imagery EEG classification, there are usually only limited amount of training samples available, therefore, RVM is more suitable when the required amount of samples is considered. Overfitting is another problem of ANN, which limits the ANN in EEG classification. Therefore, we chose RVM as the EEG signal classification algorithm in this research.

Chaos is a common phenomenon that exists in nonlinear systems. Chaos does not mean disorder, but has a delicate inner structure. Studies have shown that EEG signals possess some chaotic properties [33–39]. Furthermore, chaotic systems demonstrate rich dynamic behavior, if utilized properly in kernel based methods, which is helpful for the generalization of classifiers. Therefore, the classification capacity can be improved. Inspired by these facts and considering the flexibility of kernel function selection in RVM, a chaos kernel function for RVM is proposed in this research, and validated by 4-class MI classification.

The rest of the paper is organised as follows. In section 2, the EEG data used in the research is described and the algorithms involved in this paper are reviewed in detail. Section 3 demonstrates various experimental results. Section 4 concludes the paper with a discussion on the advantages and disadvantages of the proposed method.

## Methods

### CSP framework for feature extraction in four-class MI classification

**Fig 1:**
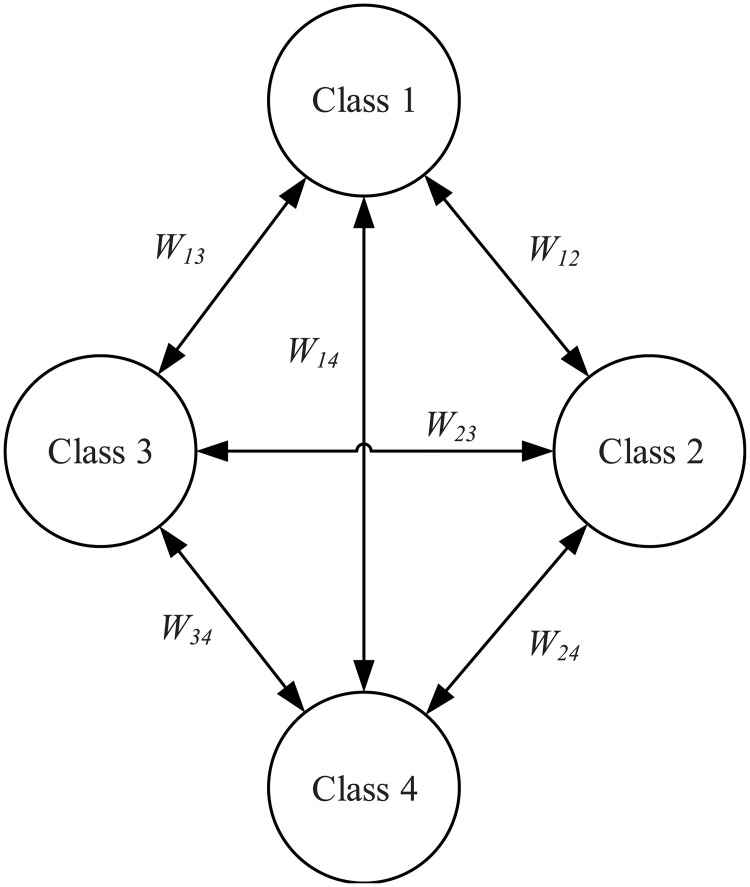
The illustration of OVO strategy [23].

Common spatial pattern (CSP) was used to extract features from the processed EEG signal [40, 41]. The CSP distinguishes two categories of samples by a spatial projection in the manner that the energy difference between the classes is maximised. For four-class classification, the OVO strategy was developed to enable CSP for the feature selection [23, 42], as illustrated in Fig 1.

For the four classes labelled as class 1, 2, 3 and 4 respectively, the OVO-CSP selects two of the four classes as the input for original CSP, which generates 6 possible selections.

Without loss of generality, for the sake of easier understanding, classification of the class 1 and class 2 is considered as an example. For the two selected classes, *X*_*i*_ (where *i*∈1, 2) denotes an EEG sample of class *i*, *X*_*i*_ is a matrix of *N* × *T*, where *N* is the number of channels, *T* is the product of sampling frequency and acquisition (seconds), that is, the number of sampling points in a channel for one MI epoch.

Dong et.al [43] demonstrated the method by decomposing the mixed spatial covariance matrix and then mapping the EEG signal to a feature space. The normalised covariance matrix of class *i* in epoch (trial) *n* is

$$R_{{}_{i,n}}^{}=\frac{X_{i}X_{i}^{T}}{trace(X_{i}X_{i}^{T})}$$ (1)

where $X_{i}^{T}$ is the transpose matrix of *X*_*i*_, and $trace(X_{i}X_{i}^{T})$ is the trace of $X_{i}X_{i}^{T}$, n = 1, 2,⋅⋅⋅, *N*_*e*_ and *N*_*e*_ is the total number of epochs for class *i*. The spatial covariance can be computed by averaging all the trials (epochs) of the class *i*.

The original EEG signal *X* is projected to the new spatial space as

Z=WX (2)

where *W* is a spatial filter calculated by CSP.

The features used for classification are obtained from (2). For each class of imagined movements, only a small amount, denoted as *m*, of the most distinguishing signal variances is selected for classification. *Z*_*k*_ (*k* = 1, 2,⋯, 2*m*) is constructed by the first *m* and last *m* rows of *Z*, which maximize the difference of variance of two-class EEG signals.

OVO-CSP transforms the four-class classification problem into six cases of two-class classification. We pick up the first and the last vectors (corresponding to the largest and the smallest eigenvalues respectively) from the sorted feature matrix *Z* as the most significant two feature vectors.

Instead of using *Z* directly, the normalised log-variances of these components are considered to be features for classification.

The feature corresponding to *Z*_*k*_ is calculated as

$$f_{k}=\text{log}(\frac{\text{var}(Z_{k})}{\sum_{i=1}^{2m}\text{var}(Z_{i})})$$ (3)

This new feature makes the distance between two classes more significant.

### RVM classification

Assume that ${\left\{u_{i}\right\}}_{i=1}^{N}$ is the eigenvector in training data and ${\left\{t_{i}\right\}}_{i=1}^{N}$ (*t*_*i*_ ∈ {0, 1}) is the corresponding target value. Then the RVM classification model can be expressed as

$$y\left(u;w\right)=\sum_{i=1}^{N}w_{i}K\left(u,u_{i}\right)+w_{0}$$ (4)

Where *K*(**u**, **u**_*i*_) is a kernel function, *w*_*i*_ is the weight of the *i*-th kernel function, **w** = [*w*_0_, *w*_1_,⋯, *w*_*N*_]^*T*^, *w*_0_ is the bias.

For the two-class classification, we adopt the Logistic Sigmoid function to map *y*(**u**; **w**) to (0, 1). Since the target value ${\left\{t_{i}\right\}}_{i=1}^{N}$ can only be 0 or 1, and each prediction is independent, the samples are assumed to be independent and identically distributed.

To avoid introducing the shortcomings similar to the SVM, such as severe over-fitting due to excessive support vectors used, the weight vector **w** is constrained with the precondition, that is, all weight vectors satisfy a zero-mean Gaussian prior distribution.

$$p\left(w|\alpha \right)=\prod_{i=0}^{N}N(w_{i}|0,\alpha_{i}^{-1})=\prod_{i=0}^{N}\frac{\alpha_{i}}{\sqrt{2\pi }}\text{exp}\left(-\frac{\alpha_{i}w_{i}^{2}}{2}\right)$$ (5)

Where *α* = [*α*_0_, *α*_1_, *α*_2_,⋯, *α*_*N*_]^*T*^ is a hyper-parameter vector which determines the prior distribution of the weight vector w, and controls the degree to which the weight deviates from its zero-mean.

Given the prior probability distribution and the likelihood distribution, the Bayes’ Rule is adopted to calculate the posterior probability of models **w** and *α* [44]

p(w,α|t)=p(w|t,α)p(α|t) (6)

In Eq (6), the posterior probability *p*(**w**|**t**, *α*) and *p*(*α*|**t**) cannot be directly solved, the approximation procedure, as used by MacKay [45], can be adopted based on Laplace’s method. And the maximum w can be calculated as follow.

$$w_{MP}^{new}=w_{MP}+\Delta w$$ (7)

$$\alpha_{i}^{new}=\frac{1-\alpha_{i}\Sigma_{i,i}}{w_{MPi}^{2}}$$ (8)

Where Δ**w** = −**H**^−1^ *g*, **H** = −(**Φ**^*T*^B**Φ** + A), $\Sigma ={\left(-H{|}_{w_{WP}}\right)}^{-1}$, **A** = *diag*(*α*_0_, *α*_1_, ⋯, *α*_*N*_), **B** = *diag*[*y*_*i*_(1 − *y*_*i*_)], *y*_*i*_ = *σ*{*y*(x_*i*_; **w**)}, *g* = **Φ**^*T*^(**t** − **y**) − A**w**, **y** = [*y*_1_, *y*_2_, ⋯, *y*_*N*_]^*T*^,

$$\Phi =[\begin{array}{ccccc} 1 & K(u_{1},u_{1}) & K(u_{1},u_{2}) & \cdots & K(u_{1},u_{N})\\ 1 & K(u_{2},u_{1}) & K(u_{2},u_{2}) & \cdots & K(u_{2},u_{N})\\ \vdots & \vdots & \vdots & \ddots & \vdots \\ 1 & K(u_{N},u_{1}) & K(u_{N},u_{2}) & \cdots & K(u_{N},u_{N}) \end{array}]$$

Σ_*i*, *i*_ is the *i*-th diagonal element in **Σ**.

The RVM algorithm model training procedure is to proceed to repeat (8), concurrent with updating (7), until some appropriate convergence conditions have been met.

In fact, with the repeated updating, the majority of *α*_*i*_ approaches infinity, and the corresponding *w*_*i*_ approaches 0. The **u**_*i*_ corresponding to the non-zero weight are relevant vectors. Assume that {u_*_} is the test sample vector, we make classification predictions by the weights obtained from the learning training data, as follows.

$$t_{*}=y\left(u_{*};w_{MP}\right)=\Phi \left(u_{*}\right)w_{MP}$$ (9)

### Chaos kernel function for RVM

**Fig 2:**
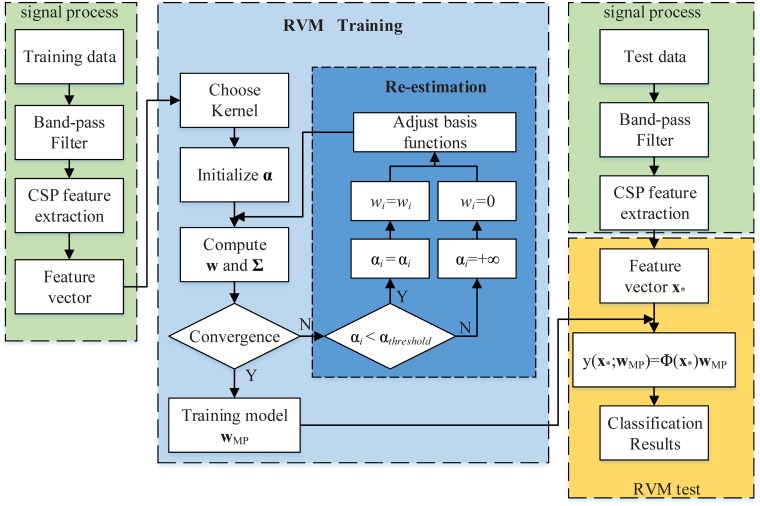
The flow of EEG signal processing and RVM algorithm.

Fig 2 roughly presents the steps of classification of BCI signals by employing the RVM. The complete procedure mainly includes four parts: training data processing, the RVM training, test data processing, and the RVM test. The re-estimation in the RVM training procedure is the key step of the algorithm to achieve sparseness.

Because the kernel functions map the feature vectors to a high-dimensional space to achieve linear classification, the properties of the kernel functions play an important role in the performance of the RVM classification algorithm. In this paper, a chaos kernel (CK) is proposed, which evolves from the probability distribution of a chaotic sequence.

**Fig 3:**
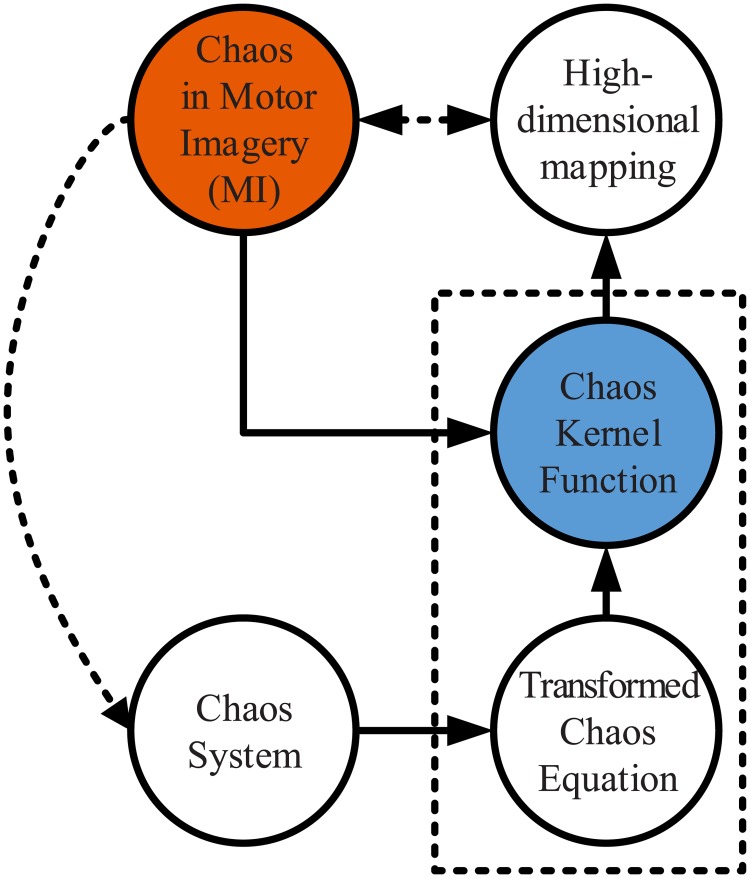
The relationship between chaos kernel and MI.

Consider the fact that the human brain signal is so complex that there is currently no theory or rule to fully explain its behaviours, but it is believed that there must be some rules behind the seemed “disorderly” signals. As shown in Fig 3, when our brain is in a state of motor imagination, the chaos in motor imagery might associate with some mental behaviours (known or unknown). The equation transformed from this chaos system can be considered to decode the brain activities. Furthermore, inspired by the idea of a kernel function, the low-dimensional collected brain signal is mapped to a high-dimensional space to find more intuitive features related to MI.

While chaos is a seemingly random irregular motion occurring in a deterministic system, it does hide a certain law. Therefore, in this paper, we are inspired to construct a kernel function for RVM from the chaos theory perspective. The Logistic Map in (10), a classic chaotic system model, is used in this paper.

$$Y_{n+1}=AY_{n}\left(1-Y_{n}\right)$$ (10)

**Fig 4:**
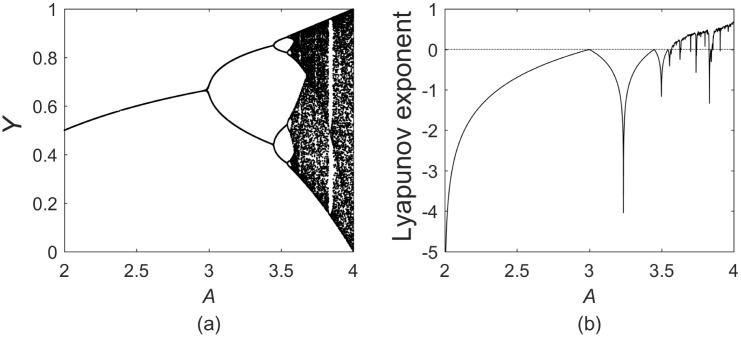
(a) Bifurcation diagram for the logistic map; (b) Lyapunov spectrum for the logistic map.

Fig 4 shows the bifurcation diagram of the typical Logistic map and the corresponding Lyapunov spectrum. When *A* = 4, the Lyapunov exponent of the Logistic mapping is more than 0, and the Logistic mapping is in a chaotic state. In this way, we think the following series of changes are based on the chaos-related equations.

When *A* = 4, the probability distribution of *Y* is

$$P\left(Y\right)=\frac{1}{\pi \sqrt{Y(1-Y)}}$$ (11)

**Fig 5:**
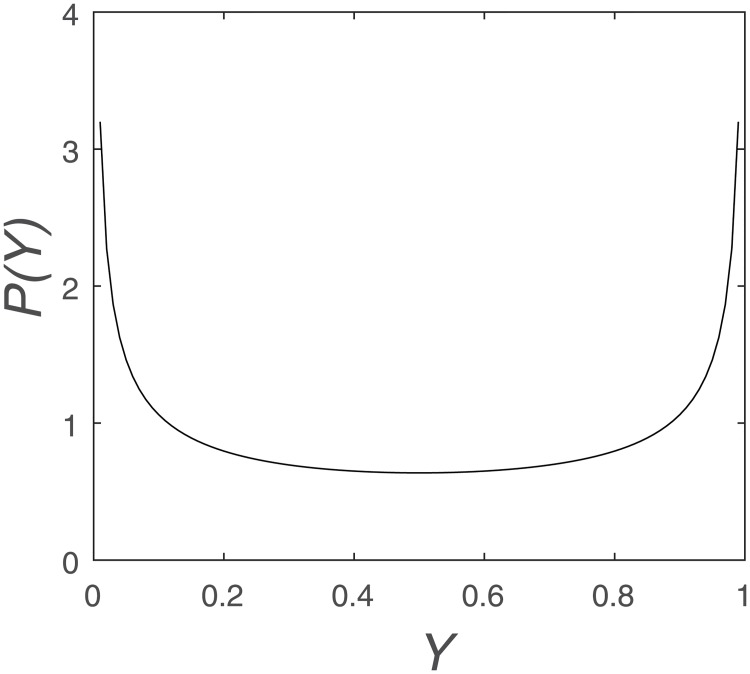
Probability distribution function of the logistic map with *A* = 4.

As shown in Fig 5, with enough iterations of the logistic map for *A* = 4, the orbit approaches arbitrarily close to every point in the interval 0<Y<1. The probability distribution function *P*(*Y*) has peaks at *Y* = 0 and *Y* = 1. But it is not very suitable for classification.

**Fig 6:**
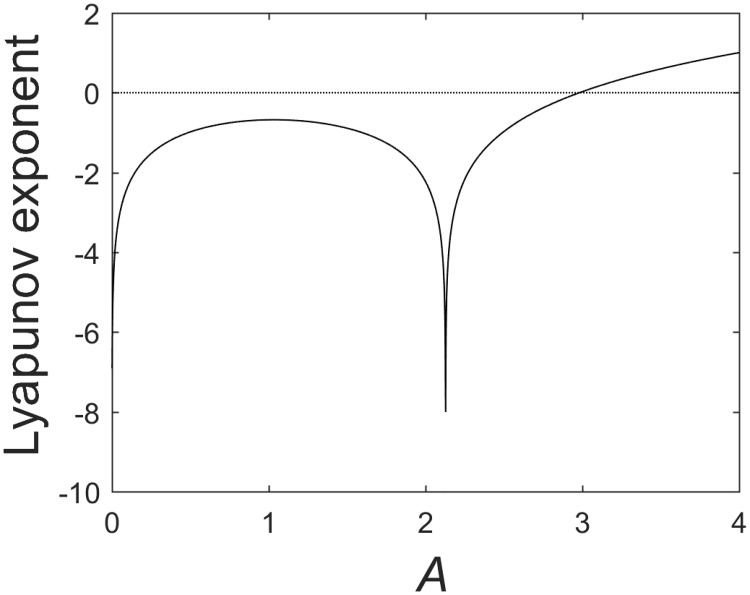
Lyapunov spectrum for the transformed system.

**Fig 7:**
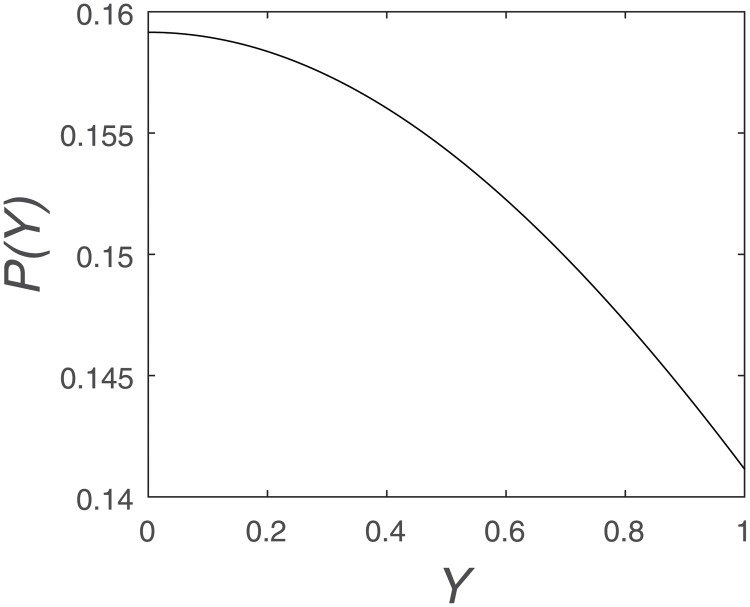
Probability distribution of *P*(*Y*) = 1/*π*(*e*^*Y*/2^+ *e*^−*Y*/2^).

In Fig 6, we can see that the Lyapunov exponent of the transformed system at *A* = 4 is greater than 0, so the system is still a chaos system. The probability distribution of the transformed chaos system is shown in Fig 7.

Applying the logit transform $f\left(Y\right)=\text{ln}\left(\frac{Y}{1-Y}\right)$ to the iterates of the Logistic Map with *A* = 4 gives a probability distribution function

$$P\left(Y\right)=\frac{1}{\pi (e^{Y/2}+e^{-Y/2})}$$ (12)

Evolve (12) into a kernel function

$$K\left(Y_{1},Y_{2}\right)=\frac{1}{\pi (\text{exp}\left(\beta ∥Y_{1}-Y_{2}∥\right)+\text{exp}\left(-\beta ∥Y_{1}-Y_{2}∥\right))}$$ (13)

where *β* is the parameter, ∥⋯∥ is the 2-norm operation.

The kernel function used in SVM has to satisfy Mercer’s condition that the kernel matrix must be a positive semidefinite matrix. While the RVM algorithm avoids this condition. Thus, the proposed chaos kernel function does not have to satisfy Mercer’s condition. Nonetheless, the kernel matrix of the chaos kernel is a positive semidefinite matrix indeed. So it can also be used in SVM.

### Four-class MI classification based on the framework of OVO-CSP

The event-related frequency bands are firstly extracted from the original EEG signals containing four-class motor imagery movements. The band-pass filter (3-24Hz) is employed, and then the filtered EEG signals are randomly divided into five groups. Four groups are used for training the classifier and the rest is for the test. Six CSP projection matrices are constructed to address the four-class classification as detailed in section 2.1, denoted as W12, W13, W14, W23, W24, and W34 respectively. Then the matrices are used to extract the features of the corresponding category from the EEG data. Finally, the six sets of features are sent to the RVM as the input vector to train six models. Using these projection matrices to extract features from the test dataset, one obtains features as the input vector of the RVM test section.

**Fig 8:**
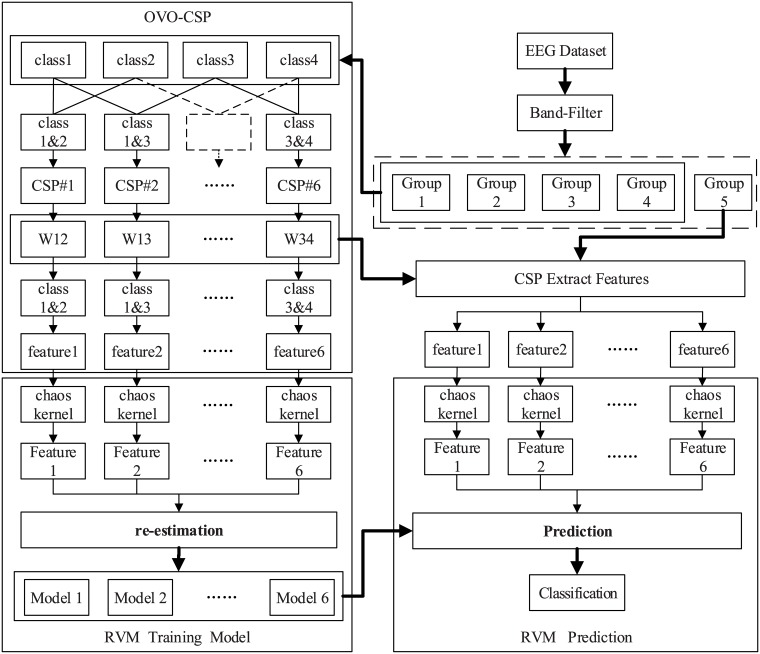
The framework of four-class MI classification based on OVO-CSP and chaos kernel RVM.

The six models obtained by the RVM training are combined with the input features of the test set to predict the classification. The whole classification procedure is shown in Fig 8. The 5-fold-cross validation is used to ensure that each group has been tested once as the test set.

## Experiments and results

### EEG dataset illustration

The dataset for the simulation experiment in this paper was derived from the BCI competition IV-II-a [46], which provided by Graz University of Technology, Austria, in 2008. The dataset contains four-class motor imagery tasks: the imagination of movement of the left hand (class 1), the right hand (class 2), both feet (class 3), and the tongue (class 4). The data recording equipment collects EEG signals and EOG signals by utilising 22 Ag/AgCl electrode channels and three monopolar EOG channels respectively, with the sampling frequency of 250 Hz. While the EOG signals included in the dataset were not used for classification in this paper, those signals provided were bandpass filtered between 0.5Hz and 100Hz. In fact, we found that only the frequency bands [3, 24] Hz change visibly during motor imagery [23]. Thus, we re-bandpass filtered the provided EEG signals with the band [3, 24] Hz.

The BCI competition 2008—Graz data set A contains two sessions on nine subjects which were recorded on two different days, taking into account the nature of unstable state of the subjects. We named the two sessions respectively T and E. Both of them have 6 runs separated by short breaks. Each run includes 48 trials (12 trials per class). That is to say, both of the sessions have 288 trials to be processed. Thus, we extracted 72 valid trials corresponding with each class of the motor imagery task. The selected four-class EEG data is re-bandpass filtered to extract features using the constructed OVO-CSP. Then five-fold cross-validation is employed to eliminate the over-fitting as much as possible. Original data (72 trials) for each category of the motor imagery tasks is randomly divided into five parts, where the four-part sample (56 trials) is used to train the RVM model and the rest (14 trials) is used for the validation. The cross-validation procedure will be repeated five times, then each part of the sample can undergo validation once.

### Results of OVO-CSP feature extraction

**Fig 9:**
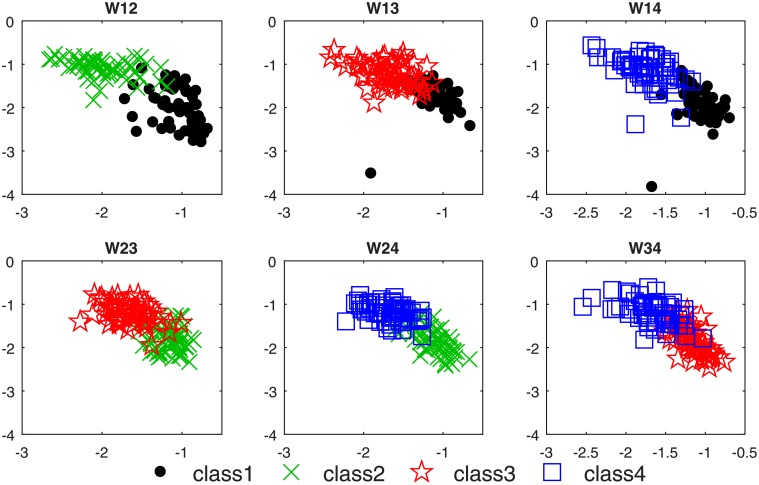
The distribution of most significant feature vector pairs obtained by OVO-CSP.

The four-class MI classification is transformed into six cases of two-class classification by OVO-CSP. The results of the feature extraction are depicted in Fig 9, showing the distribution of the most significant feature vector pairs obtained by OVO-CSP. Fig 9 suggests that the OVO-CSP obtains separable feature distributions used for RVM classification.

### Results and comparison with existing methods

To illustrate the performance of the proposed kernel function, the Gaussian kernel and polynomial kernel, shown in (14) and (15) respectively, are considered for comparison.

$$K\left(Y_{1},Y_{2}\right)=\text{exp}\left(-\frac{∥Y_{1}-Y_{2}∥}{2\sigma^{2}}\right)$$ (14)

where *σ* is the width parameter.

$$K\left(Y_{1},Y_{2}\right)={\left[Y_{1}{\left(aY_{2}\right)}^{T}+1\right]}^{d}$$ (15)

where *a* is a user-specified scalar parameter, and the polynomial degree *d* chosen in this paper is 2.

Comparison of two sessions’ classification accuracy about Polynomial kernel function (PK), Gaussian kernel function (GK), and the proposed kernel function (chaos kernel function, CK) are shown in Tables 1 and 2. Each session was randomly divided into five parts (each part contains 56 epochs), four were selected for the training weight model, and the remaining one for verification. Five cross validation ensures that every part will be validated. Thirty experiments were conducted in order to gain reliable results. The average accuracy and standard deviation are calculated.

**Table 1:** Comparison of 3-kind kernels based on RVM on session T.

Subject	Polynomial kernel (mean ±std%) (*a* = 1.26, *d* = 2)	Gaussian kernel (mean ±std%) (*σ* = 0.95)	Chaos kernel (mean ±std%) (*β* = 0.5)
**S1**	69.4±1.7	68.9±1.5	**69.7**±**1.2**
**S2**	47.3±1.8	46.5±2.0	**48.6**±**1.5**
**S3**	**76.9**±**1.1**	75.9±1.3	76.4±0.8
**S4**	52.1±1.3	51.0±2.0	**52.9**±**1.2**
**S5**	38.7±2.2	38.7±2.1	**38.9**±**1.8**
**S6**	41.5±2.2	**41.7**±**1.5**	40.7±1.1
**S7**	72.9±1.5	73.2±2.2	**73.2**±**1.0**
**S8**	80.2±1.1	79.8±1.6	**80.7**±**0.7**
**S9**	73.3±1.2	72.9±0.8	**73.6**±**0.9**
**Average**	61.4±15.4	60.9±15.3	**61.6**±**15.4**

**Table 2:** Comparison of 3-kind kernels based on RVM on session E.

Subject	Polynomial kernel (mean ±std%) (*a* = 1.26, *d* = 2)	Gaussian kernel (mean ±std%) (*σ* = 0.95)	Chaos kernel (mean ±std%) (*β* = 0.5)
**S1**	67.8±1.5	68.2±1.4	**69.6**±**1.4**
**S2**	**44.2**±**1.9**	43.6±1.6	44.1±1.3
**S3**	78.9±1.4	79.0±1.3	**80.4**±**1.0**
**S4**	**59.8**±**1.9**	59.5±2.1	59.6±1.9
**S5**	49.3±1.9	48.7±2.0	**50.4**±**1.5**
**S6**	**44.2**±**1.3**	43.7±1.1	43.9±1.3
**S7**	75.7±1.8	75.7±1.7	**76.4**±**1.5**
**S8**	81.3±1.6	80.9±1.6	81.4±1.2
**S9**	83.1±1.2	**83.2**±**1.0**	**83.2**±**0.9**
**Average**	64.9±15.1	64.7±15.3	**65.4**±**15.3**

Table 1 shows that the average accuracy of classification of the three kernel functions (PK, GK and CK) is 61.4 ± 15.4%, 60.9 ± 15.3% and 61.6 ± 15.4%, respectively. The overall performance of CK is better than PK and GK. In Table 1, each subject’s classification result is made up of two parts, the average accuracy and standard deviation respectively. They are two indicators in statistics. The smaller the standard deviation is, the more the statistical results are concentrated on both sides of the mean (i.e., the average accuracy). It can be seen from the Tables 1 and 2 that the standard deviation of classification results for the chaos kernel RVM are generally smaller than the others, indicating that the results are more centralized and more credible. Which suggested the proposed method is more effective for EEG signal classification.

For the individual subject case, the best of the three kernel functions are bolded. In most cases, subjects 1, 2, 4, 7, 8 and 9, the proposed chaos kernel function achieved a higher accuracy. For the remaining subjects, the proposed chaos kernel function yields a slightly lower accuracy.

Similar results are presented in Table 2 for the second session, in which the chaos kernel performance performed better on subjects 1, 3, 5, 7, 8 and 9 than with the other kernels. The polynomial kernel function performed better for subjects 2, 4 and 6. The chaos kernel function achieves better accuracy with 65.4 ± 15.3%, a little advantage over the other by 64.9 ± 15.1%, 64.7 ± 15.3%, respectively.

**Fig 10:**
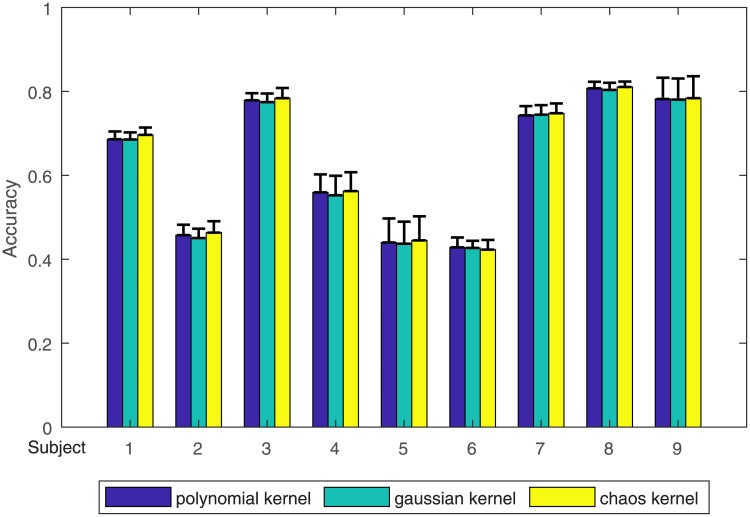
The overall classification result on two sessions.

Fig 10 exhibits the final results of 3 kernel functions in each subject. The value of the accuracy and standard deviation are computed by the datum from Tables 1 and 2. Except for subject 6, the result achieved by the proposed kernel function is better than that obtained by the other two kernel functions. The best accuracy is 81.05%, obtained by subject 8 using the chaos kernel.

Table 3 presents the comparison between the RVM algorithm, based on three kernels, and SVM. All the computations are carried on a Lenovo computer (CPU 3.3 GHz) with the software Matlab (2015b).

**Table 3:** Comprehensive comparison of RVMs based on three kernels and SVM.

	kernels	Processing time for training procedure (s)	Processing time for training procedure (s)	RVs or SVs	Accuracy (%)
**RVM**	Polynomial kernel (a = 1.26; d = 2)	0.8767	0.0074	16	63.1±15.2
	Gaussian kernel (*σ* = 0.95)	1.2189	0.0067	17	62.8±15.3
	Chaos kernel (*β* = 0.5)	1.3899	0.008	13	63.6±15.4
**SVM**	Polynomial kernel (*g* = 3.0314)	7.2395	0.005	183	64.5±15.7

Table 4 presents the comparison between the proposed method and the competition methods [47]. We can see that the main difference between our method and the second method is the difference of classifiers, however, the results are very close. The result of the proposed method is obviously more effective than the third, fourth and fifth methods.

**Table 4:** Comparison of the proposed method and the competition methods.

Methods	Propressing	Features	Classification	Kappa
**1**	bandpass filter (4-40Hz)	OVR-FBCSP	Naive Bayes Parzen Window classifier	0.57
**2**	bandpass filter (8-30Hz)	OVO-CSP	LDA & Bayesian classifier	0.52
**3**	bandpass filter (8-25Hz)	CSP	Two-hierarchical SVM classifier	0.31
**4**	NTSPP+CSP	CSP	LDA & SVM	0.30
**5**	bandpass filter (8-25Hz)	CSP	Two-hierarchical SVM classifier	0.29
**Proposed method**	bandpass filter (3-24Hz)	cmOVO-CSP	RVM	0.515

**Fig 11:**
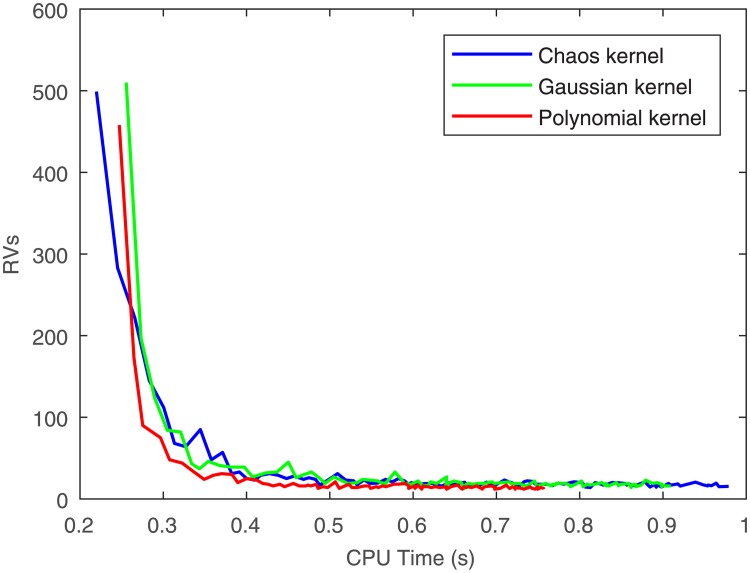
The relationship of RVs and CPU time.

It is evident in Fig 11 that at 0.6s, the polynomial kernel RVM and the chaos kernel RVM converge, and the Gaussian kernel RVM converges at 0.65s. They yield almost the same convergence rate.

**Fig 12:**
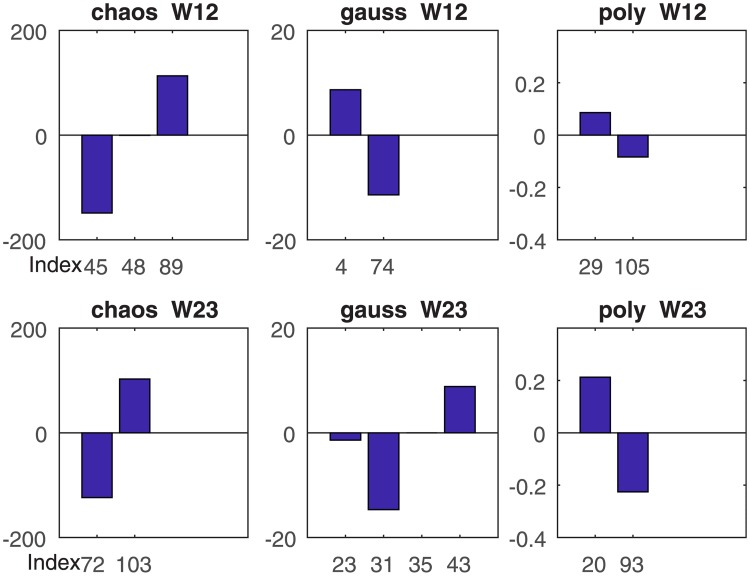
Comparison of two typical RVM training weights of three kernel functions.

Fig 12 depicts the trained weights computed by three RVM kernel functions. The horizontal axis denotes the index of the RVs corresponding to the learned weight. There are no more than four learned weights in each graph, which produces the sparse classification results. The vertical axis denotes the value of the learned weight. The value of the learned weights in the different kernel functions varies so greatly, up to orders of magnitude. This is so because those weights are computed by the corresponding kernel function. While we pay attention to the difference between the positive and negative weights in each graph, which is the key indicator to distinguish the features, it is obvious that the greater the difference, the easier it is to distinguish the two-class signals.

**Fig 13:**
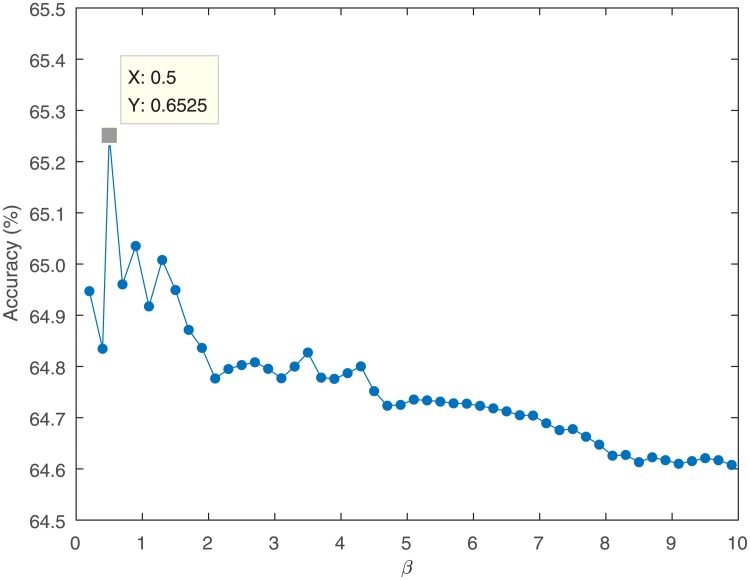
The influence of parameter *β* on the accuracy of classification.

Fig 13 shows the influence of the parameter beta, in the chaos kernel, on the classification results. It can be seen from the Fig 13 that the overall trend is that as the value of the parameter beta becomes larger, the classification accuracy is decreased, while at the point of *β* = 0.5, we get the best classification accuracy.

## Discussion and conclusion

In this paper, a new chaos kernel was proposed for relevance vector machine to classify four-class EEG of motor imagery. The raw EEG signals are addressed by 3-24 Hz band-pass filter to remove artifacts and uncorrelated frequency bands. And the four-class classification problem is transformed into six two-class problem under the framework of OVO-CSP method. Then the feature vectors extracted by OVO-CSP are sent to the RVM for classification.

Compared with SVM, RVM has a significant advantage in training time and the number of relevance vectors (or support vector for SVM), as seen in Table 3. If the number of RVs or SVs is smaller, the classification model will be simpler. Especially, as the input samples increase, the complex classification model will produce a huge amount of computation, resulting in slow classification. At the same time, the cost of smaller RVs is that the classification accuracy is slightly lower (see Table 3), thus it is also evident that the classification accuracy of RVM is not as good as that of the SVM. Although the test time of the RVM is slightly longer than that of the SVM, it requires a much shorter training time than the SVM.

The proposed kernel function evolved from the distribution function of a chaos system. For a long time, researchers have been studying the phenomenon of chaos in the brain. The EEG signal sometimes appears as a chaotic phenomenon when the neural network changes from one sequential structure to another [38]. Furthermore, the pioneers have proved that the EEG signal is controlled by several independent dynamic variables. This is very similar to the production of a chaotic system. Thus, we boldly predict that there more features of the EEG may be found by using chaos theory.

Although the proposed kernel function does not have significant advantages compared with the Gaussian and Polynomial kernel functions, it suggested another approach for EEG signal analysis, which is different from the classic SVM method. In the future, further attempts will be made to find a more suitable kernel function that stems from a chaos system.

## Supporting information

S1 Table Data for drawing Fig 5. Data for probability distribution of the logistic map with *A* = 4.
(XLSX)

S2 Table Data for drawing Fig 6. Data for describing the Lyapunov exponent of the transformed system with *A*.
(XLSX)

S3 Table Data for drawing Fig 7. Data for describing the probability distribution of the transformed chaos system.
(XLSX)

S4 Table Data for drawing Fig 9. Data for describing the results of the feature extraction in Fig 9.
(XLSX)

S5 Table Detailed data of the results in Table 1 (session T). This is the detailed result of 30 experiments per subject and Table 1 is the result of averaging 30 experiments.
(XLSX)

S6 Table Detailed data of the results in Table 2 (session E). This is the detailed result of 30 experiments per subject and Table 2 is the result of averaging 30 experiments.
(XLSX)

S7 Table Data for drawing Fig 10. Average accuracy and standard deviation of two sets (Tables 1 and 2) of data for 9 subjects.
(XLSX)

S8 Table Data for drawing Fig 11. The data for describing convergence curves of three kernels in Fig 11.
(XLSX)

S9 Table Data for drawing Fig 12. The data of the trained weights computed by three RVM kernel functions.
(XLSX)

S10 Table Data for drawing Fig 13. The detailed data for explain the influence of the parameter beta, in the chaos kernel, on the classification results.
(XLSX)
